# Polyclonal Regulatory T Cell Manufacturing Under cGMP: A Decade of Experience

**DOI:** 10.3389/fimmu.2021.744763

**Published:** 2021-11-18

**Authors:** Joanna Balcerek, Brian R. Shy, Amy L. Putnam, Lisa M. Masiello, Angela Lares, Florinna Dekovic, Luis Acevedo, Michael R. Lee, Vinh Nguyen, Weihong Liu, Sreenivasan Paruthiyil, Jingying Xu, Ashley S. Leinbach, Jeffrey A. Bluestone, Qizhi Tang, Jonathan H. Esensten

**Affiliations:** ^1^ Department of Laboratory Medicine, University of California San Francisco, San Francisco, CA, United States; ^2^ Diabetes Center, University of California San Francisco, San Francisco, CA, United States; ^3^ Department of Surgery, University of California San Francisco, San Francisco, CA, United States; ^4^ Sean N. Parker Autoimmune Research Laboratory, University of California San Francisco, San Francisco, CA, United States

**Keywords:** regulatory T cell manufacturing, cGMP, *ex vivo* expansion, cellular therapy, regulatory T cells

## Abstract

We report on manufacturing outcomes for 41 autologous polyclonal regulatory T cell (PolyTreg) products for 7 different Phase 1 clinical trials over a 10-year period (2011-2020). Data on patient characteristics, manufacturing parameters, and manufacturing outcomes were collected from manufacturing batch records and entered into a secure database. Overall, 88% (36/41) of PolyTreg products met release criteria and 83% (34/41) of products were successfully infused into patients. Of the 7 not infused, 5 failed release criteria, and 2 were not infused because the patient became ineligible due to a change in clinical status. The median fold expansion over the 14-day manufacturing process was 434.8 -fold (range 29.8-2,232), resulting in a median post-expansion cell count of 1,841 x 10^6^ (range 56.9-16,179 x 10^6^). The main correlate of post-expansion cell number was starting cell number, which positively correlates with absolute circulating Treg cell count. Other parameters, including date of PolyTreg production, patient sex, and patient age did not significantly correlate with fold expansion of Treg during product manufacturing. In conclusion, PolyTreg manufacturing outcomes are consistent across trials and dates of production.

## Introduction

Regulatory T cells (Tregs) are a subset of CD4^+^ T cells that suppress excessive immune activation and prevent autoimmunity (1–4). Adoptive cell therapy with polyclonal Tregs (PolyTregs) shows a favorable safety profile in patients with autoimmune disease, solid organ transplant, and graft versus host disease (5–13).

Efficient clinical-scale manufacturing of Treg products requires isolation of Tregs and adequate *ex-vivo* expansion while maintaining Treg cell identity. Several different clinical scale methods for Treg product manufacturing have been published that use different Treg sources, isolation methods, expansion methods, and dose formulations (14). However, the overall approach to *in vitro* Treg manufacturing is broadly similar. Most Treg manufacturing approaches start with autologous patient peripheral blood, which contains a small percentage of Tregs. Tregs are then selected by one or more methods such as magnetic-activated cell sorting (MACS) for CD25^+^ cells or fluorescence activated cell sorting (FACS) for CD4^+^CD25^+^CD127^lo/-^ cells (6–8, 11–18). Tregs are then activated with potent stimulation through the T cell receptor and CD28 in the presence of high levels of exogenous interleukin-2 (IL-2). Beads conjugated with anti-CD3 and anti-CD28 are commonly used, although other activation reagents such as stimulated B cells or artificial APCs have also been described (19, 20). Rapamycin is sometimes added to the culture to prevent outgrowth of contaminating effector cells. Expansion methods vary greatly in the cell culture media, length of culture, and type of antigen receptor stimulus, and frequency of that stimulus. Most Treg manufacturing methods have been developed and validated using blood from healthy donors or with blood from a single patient population.

Here we describe our experience manufacturing autologous polyclonal Treg products for patients with autoimmune diseases or transplantation in multiple clinical trials over a 10-year period. The approach to Treg manufacturing used for these patients involves sorting of CD4^+^CD25^+^CD127^lo/-^ Treg from peripheral blood mononuclear cells using FACS, following by expansion for 14 days in medium containing IL-2. Stimulation with anti-CD3/CD28 beads is provided on days 0 and 9 of the expansion. These data provide insight into the effects of intrinsic patient variability and patient disease status on Treg manufacturing outcomes.

We provide data on 41 *in vitro* expanded polyclonal CD4^+^CD127^lo/‐^CD25^+^ PolyTreg products manufactured for 7 clinical trials in patients with autoimmune conditions, patients who have undergone kidney transplantation, or patients receiving *de novo* pancreatic islet transplant. We quantify manufacturing outcomes and describe significant correlations using detailed records of patient characteristics, manufacturing parameters, and cell manufacturing outcomes.

## Materials and Methods

### Manufacturing of Treg Products for Clinical Trials

**Figure 1:**
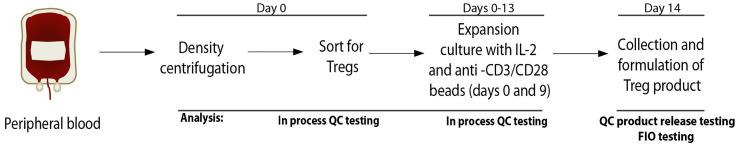
Outline of Treg cell collection and manufacturing process. QC, quality control; FIO, for information only.

Isolation, *ex-vivo* expansion, and quality control testing of Treg products was performed at the Human Islet and Cellular Transplantation Facility (HICTF) and GMP Facility, an FDA-registered cellular therapy facility at the University of California San Francisco (UCSF), using methods as previously described (21). Briefly, peripheral blood was collected in Anticoagulant Citrate Phosphate Dextrose Solution, USP (CPD) Blood Pack Units (Fenwal, Lake Zurich, IL) and processed within approximately 24 hours of collection. Peripheral blood mononuclear cells (PBMCs) were collected following density gradient centrifugation using Ficoll-hypaque solution (Amersham/GE Healthcare, Piscataway, NJ). Cell number and viability were assessed. Tregs were then isolated by fluorescence activated cell sorting (FACS) using fluorescent labeled anti-CD4, -CD127 and -CD25 antibodies. Cells were gated on CD4^+^ CD25^+^ CD127^lo/-^. An aliquot of sorted cells was run through the flow cytometer again to assess purity. Following isolation, Tregs were plated at 0.25 x 10^6^ cells per mL in a 24-well plate (Thermo Fisher; Waltham, MA) in X-VIVO 15 media (Lonza) containing 10% human AB serum from qualified donors. Tregs were activated with Dynabeads ClinExVivo anti-CD3/CD28 coated microbeads (Invitrogen; Carlsbad, CA) at a 1:1 bead to cell ratio. On day 2 of culture, the culture volume was doubled and IL-2 (Proleukin; Chiron Therapeutics, Emeryville, CA and others) was added to a final concentration of 300 IU/mL. Cells were resuspended, counted and fresh media containing IL-2 was added on days 5, 7, 9 and 12. IL-2 was maintained at 300 IU/mL assuming total consumption from media at each feeding. On days 5, 7, and 9, cell concentration was maintained between 0.2 x 10^6^ and 0.3 x 10^6^ cells per mL in appropriately sized plastic plates or flasks (Costar; Cambridge, MA). On day 12, cell concentration was maintained at 0.5 x 10^6^ in culture bags (Saint-Gobain; Gaithersburg, MD), cell number permitting. On day 9, cells were restimulated with fresh anti-CD3/CD28 coated beads at a 1:1 ratio (**Figure 1**). Cells were harvested on day 14, counted, and analyzed for CD4, CD8, CD25 and FOPX3 expression using flow cytometry. Treg specific demethylated region (TSDR) methylation status was determined by Epiontis (Berlin, Germany). Products meeting release criteria were released for infusion. Method adjustments over the period described in this study included: qualification and use of new lots of human serum when the initial lots were used up, and a change in release criteria. The release criterion for CD8^+^ cells was changed from <5% CD8^+^ to <5% CD8^+^CD4^-^ since we occasionally observed CD4^+^FOXP3^+^ Treg that expressed low levels of CD8. These cells were present in only a small subset of patients and may reflect an underlying genetic de-repression of CD8 in cells committed to the CD4 lineage.

### FOXP3 Locus Methylation Analysis

TSDR methylation assays were performed by Epiontis using an established protocol (22). In brief, methylation status at the FOXP3 enhancer CNS2 region was determined using bisulfite treatment of genomic DNA extracted from Treg product cell samples, followed by real-time PCR analysis using methylation-specific primers. The percentage of unmethylated DNA was calculated using the following formula: unmethylated DNA/(unmethylated DNA + methylated DNA). For female patients, the results were multiplied by 2 due to the presence of a fully methylated, inactivated X chromosome in female patients.

### Data Collection and Storage

Manufacturing batch records from each Treg product were obtained and data were manually extracted and recorded in Research Electronic Data Capture (REDCap, v9.5.25), a secure and Health Insurance Portability and Accountability Act–compliant web-based system for building and managing surveys and databases.

### Analysis

Means of continuous variables were compared using ANOVA and Kruskall-Wallis test. Where only two categories existed, means were compared by Student’s two-tailed t-test. Linear regression models were constructed for assessment of correlation between variables. For categorical variables, post-expansion cell number was classified by level and means were compared using ANOVA and Kruskall-Wallis test. All data analysis was performed using Prism (v9.1.0, GraphPad Software).

Clinical Trials for which Treg products were manufactured are: NCT01210664 (T1D), NCT 02772679 (TILT), NCT02428309 (ALE08; “Lupus”), NCT03239470 (APG01; “Pemphigus”), NCT02088931 (TASKp), NCT02711826 (TASK), NCT03444064 (pTregITX).

## Results

**Figure 2:**
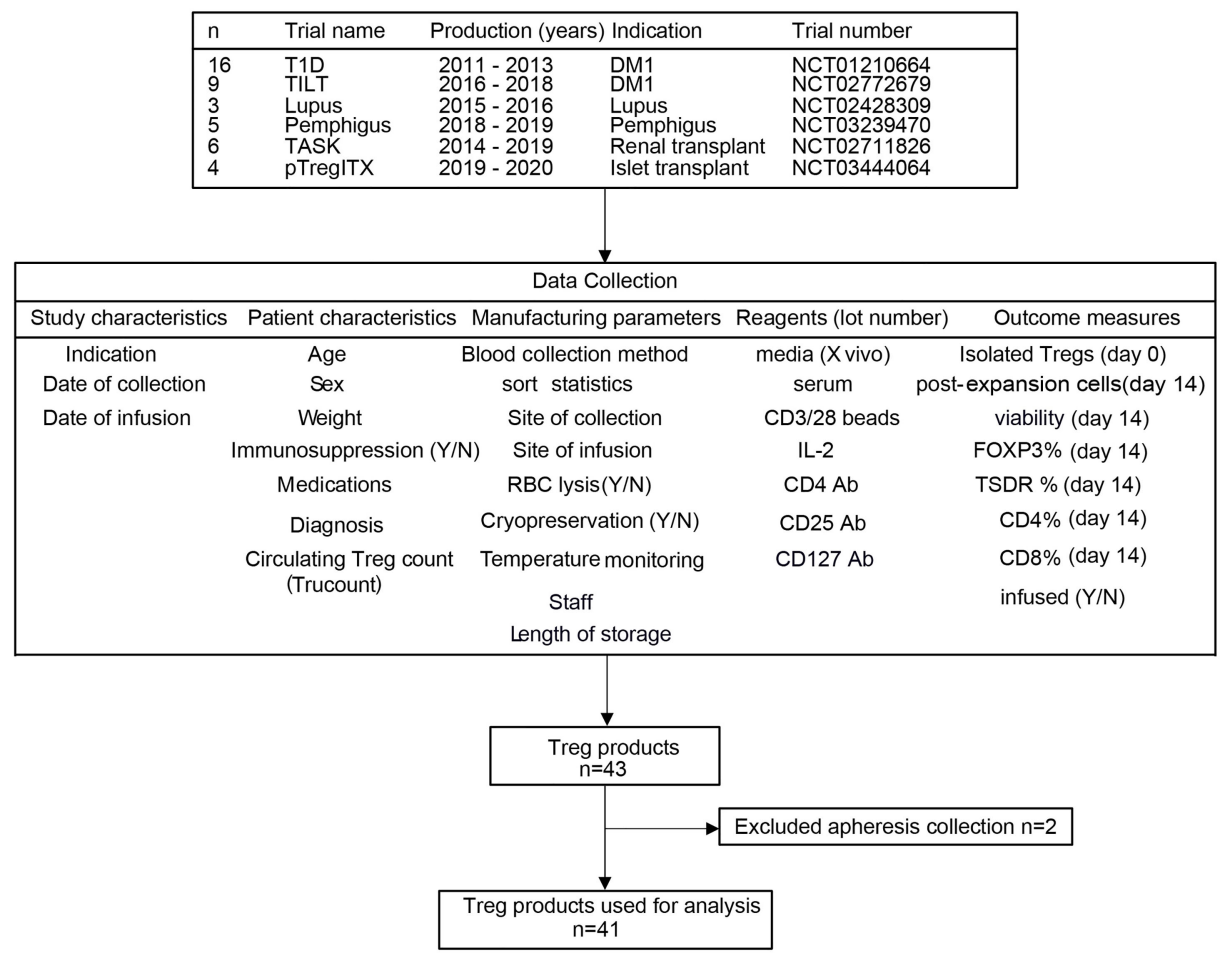
Study diagram. Schematics of PolyTreg clinical trials, data collection, and analysis.

Autologous polyclonal Treg products manufactured for clinical trials between 2011 and 2020 were included in this analysis. During that period, a total of 43 polyclonal Treg products were manufactured for 7 different clinical trials. **Figure 2** includes the trial NCT numbers, the years of production, and the clinical indication for enrollment in the clinical trial. Data extracted from manufacturing batch records were entered into a secure database. Data analyzed included study characteristics, patient characteristics, manufacturing parameters, reagent lots and outcome measures. Two products were excluded from further analysis because they were manufactured from autologous apheresis collections and were therefore not comparable to all the other products, which were manufactured from whole blood.

Key manufacturing parameters including the number of Treg isolated at the beginning of the manufacturing process, fold expansion of Treg during manufacturing, and percentage of FOXP3^+^ cells by flow cytometry in the final product are summarized in **Table 1**.

**Table 1:** Manufacturing outcomes of PolyTregs by infused (first column), not infused (second column) or total (third column) products.

	PolyTreg manufactured		Total
	PolyTreg infused	PolyTreg not infused	
Number products	34	7	41
Age (min-max)	40 (22-69)	47.8 (41-64)	42 (22-69)
Sex: F, M	12, 22	4, 3	16, 25
Indication			
Type 1 DM	23	2	25
Lupus	1	2	3
Pemphigus	4	1	5
Renal transplant (TASK)	4	0	4
Allogeneic islet transplant	2	2	4
			
Treg/µL blood (Trucount) (min-max)	56.9 (23.0-115.5)	43.4 (32.4-64.8)	56.1 (23.0-115.5)
Isolated Treg number (x10^6^) (min-max)	4.3 (1.1-11.8)	1.6 (0.7-7.8)	4.2 (0.7-11.8)
Isolated Treg purity % (min-max)	99.0 (96.3 - 99.9)	99.2 (97.6-100)	99.1 (96.3-100)
Post-expansion Treg number (x10^6^) (min-max)	2031 (186-12920)	215 (37.9-16179)	1841 (56.9-16179)
Fold expansion (min-max)	511.5 (29.8-1704)	150.7 (37.9-2232)	434.8 (29.8-2232)
Final CD4^+^ % (min-max)	97.1 (95.0-98.9)	95.6 (86.6-98.5)	96.8 (86.6-98.9)
Final FOXP3^+^ % (min-max)	94.0 (73.0-98.6)	90.2 (20.9-93.3)	93.9 (20.9-98.6)
Final CD8^+^ % (min-max)	0.31 (0.03-3.3)	0.91 (0.1-7.6)	0.37 (0.03-7.6)

Number of products is indicated in following rows: number products, sex, and indication. Remaining rows indicate median, with minimum and maximum values in parentheses.

**Figure 3:**
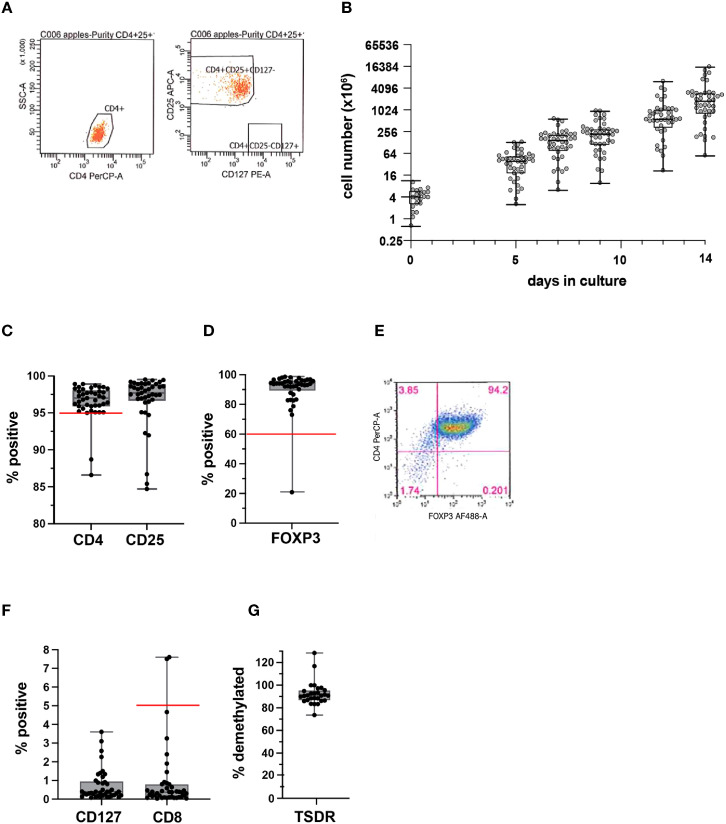
Quality assessment of isolated Tregs and PolyTreg products. Representative flow cytometric plots of post-sort purity check **(A)**. Cell numbers over the 14-day manufacturing period for all products (n=41) **(B)**. Post-expansion % CD4+, CD25+ **(C)**, and FOXP3^+^ in PolyTreg products analyzed by flow cytometry **(D, E)**. CD127, or CD8 expression in Treg products by flow cytometry **(F)**. TSDR methylation of PolyTreg products, expressed as % methylated sites **(G)**. Release criteria cutoffs are highlighted as a red line in each plot, as applicable.

The median peripheral blood Treg number in patient blood was 56.1 cells/mL (range 23.0-115.5). A median of 462.5 mL (range: 367-532.5) of whole blood was used as a starting material. Peripheral blood mononuclear cells (PBMCs) were isolated by Ficoll density gradient, then stained with GMP-grade fluorochrome-conjugated anti-CD4, anti-CD25, and anti-CD127 antibodies, and CD4^+^CD25^+^CD127^lo/-^Tregs were then sorted using FACS. The sorts yielded a median of 4.2x10^6^ Tregs (range: 0.7-11.8). Median post-sort purity was 99.1% (range: 96.3-100), based on the markers used for sorting. A representative purity check is shown (**Figure 3A**). Median fold expansion during the 14-day manufacturing period for all products was 434.8-fold (range: 29.8-2232). This fold expansion was sufficient for infused products to meet the minimum required dose, as defined by the various clinical trial protocols. Median cell viability post-expansion was 99.3% (range: 90.5-100). We did not have any product failures due to low Treg viability.

Cell growth over the 14-day manufacturing period for all 41 PolyTreg products is shown (**Figure 3B**). Flow cytometry analysis of the final Treg product showed a median of 96.8% (range: 86.6-98.9) CD4^+^ (>95% required for product release, **Figure 3C**), 93.9% (range: 20.9-98.6) FOXP3^+^ (>60% required for product release, **Figure 3D**) with representative flow cytometry (**Figure 3E**), and 0.37% (range: 0.03-7.6) CD8^+^ cells (<5% required for product release, **Figure 3F**). Methylation analysis of intron 1 of the *FOXP3* gene shows consistently high levels of demethylation (median 90.8%, range: 73.62-128.4), which confirms the identity of the cells as Treg (**Figure 3G**). Calculated demethylation results were slightly over 100% in a few samples. These were all samples from female patients having more than 50% demethylated TSDR resulting in >100% after doubling to account for X chromosome inactivation. The higher than 50% demethylation in female samples may be due to over conversion of methylated cytosine to uracil in these samples.

Out of a total of 41 PolyTreg products produced, 34 were infused (34/41 = 83%). Of the infused products, the leading indication was Type 1 diabetes mellitus, totaling 23 products. Five infused products were manufactured for other autoimmune indications (systemic lupus erythematosus, pemphigus vulgaris), and 6 were made for patients with solid organ or tissue transplant (kidney, islet transplant). The remaining 7 products were not infused. Of the products not infused, 4 did not meet release criteria. Specifically, products were not released due to low percentage of CD4^+^ cells (n=2), of which one also had a high percentage of CD8^+^ T cells. One additional product had a low percentage of FOXP3^+^ cells (n=1) in the final product. These manufacturing failures were due to outgrowth of non-Treg T cells in the cultures. The fourth product that did not meet release criteria contained 7.36% CD4^+^CD8^+^ cells that were later determined to be FOXP3^+^ and suppressive *in vitro*. Subsequently the release criterion was changed from <5% CD8^+^ to <5% CD8^+^CD4^-^ to account specifically for contamination by non CD4-expressing cells. Finally, one product was not infused due to a false positive in-process fungal culture, which was later determined to be due to a microbiology lab error. Overall, 88% (36/41) of products met release criteria.

**Figure 4:**
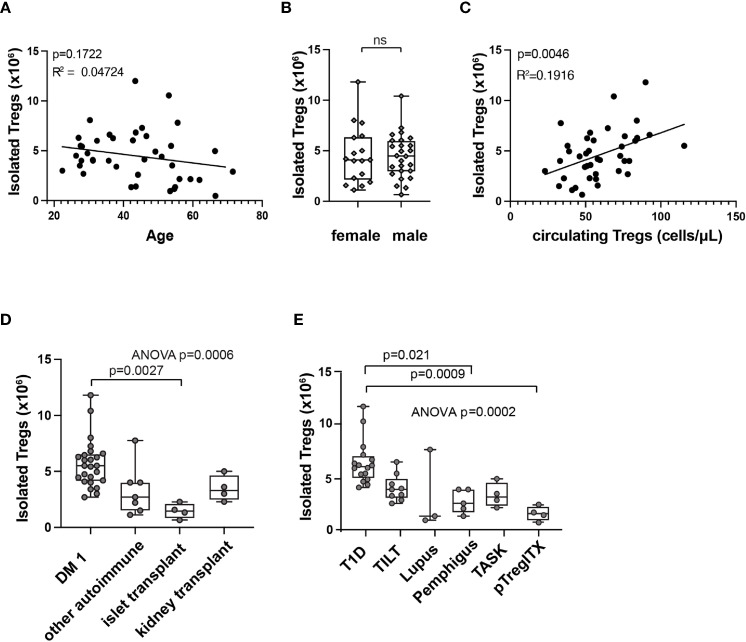
Correlations with isolated Treg numbers at start of manufacturing (day 0). Correlation between patient age **(A)**, sex **(B)**, or circulating Treg number **(C)** and isolated Treg number on day 0 of manufacturing by simple linear regression. R squared (R^2^) values and p-value assess goodness of fit, and significance of slope not equal to zero. Isolated Treg number on day 0 of manufacturing, subcategorized by patient indication **(D)** or by clinical trial **(E)**. Means comparison, ns, not significant. In box and whisker plots, the box shows mean and quartiles, whiskers show minimum and maximum values.

Interestingly, we observed a high variability in Treg product yield (**Figure 3B**). We thus explored to determine if patients and manufacturing parameters may be associated with product yield. There was no clear correlation between the number of Treg isolated from the starting material and patient age (**Figure 4A**). Treg numbers isolated from starting material were similar between male and female patients (**Figure 4B**).

A modest correlation between circulating Treg number in patients’ whole blood and Treg number isolated at the beginning of manufacturing (**Figure 4C**) was observed. Further analysis of different patient populations showed significant differences in the number of Treg isolated from starting material between patients with different indications for Treg therapy (**Figure 4D**). These results may reflect differences in previous treatment or in disease state that could affect Treg numbers in the peripheral blood. When analyzed by individual trial, initial isolated Treg number differed by trial and patient population (**Figure 4E**).

**Figure 5:**
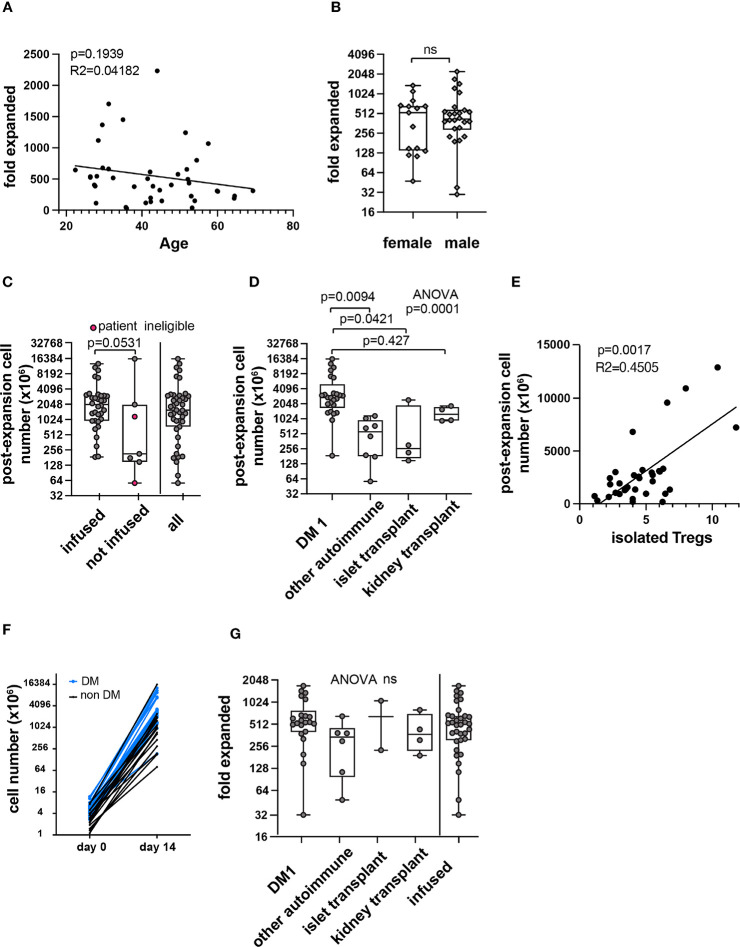
Correlations with PolyTreg products *in-vitro* expansion. Correlation between age **(A)** or sex **(B)** and Treg fold expansion. Post expansion Treg number in all products (n=41) in millions by infusion status or total **(C)**. Pink circles in the second column indicate patient ineligibility for infusion. The remaining data points in the “not infused” column represent manufacturing failures. Post-expansion cell number subcategorized by indication (n=41) **(D)**. Post-expansion cell number plotted against isolated Treg number **(E)** with simple linear regression (n=36). Isolated and post-expansion cell number plotted individually by patient (n=36) **(F)**. Blue lines and symbols represent patients with type 1 diabetes, black lines and symbols represent all other indications. Fold-expansion by indication or among all infused products (n=36) **(G)**. R squared (R^2^) values and p-value assess goodness of fit, and significance of slope not equal to zero. In box and whisker plots, the box shows mean and quartiles, whiskers show minimum and maximum values. ns, not statistically significant.

We then sought to explore associations with total cell yield at the end of manufacturing. Age and sex were not associated with a statistically significant difference in Treg fold expansion (**Figures 5A, B**). Post expansion cell number generally corresponded with different trials and populations (**Figures 5C, D**). Because products failing release criteria contained contaminating cells, post-expansion cell number and fold-expanded values would be skewed and therefore these datapoints were excluded from further analysis. The remaining products were analyzed for significant associations (n=36). A significant correlation was observed between Treg number isolated at the start of manufacturing and post-expansion cell yield (**Figure 5E**). The five data points with the highest post-expansion cell yield for isolated Treg number were from type 1 diabetes patients. Consistent with this, individually plotted isolated and post-expansion cell numbers show a generally similar trajectory with highest cell yield from type 1 diabetes patients (**Figure 5F**).

There was marked variability in Treg number sorted from patient blood (**Figure 4C**). To normalize for isolated cell number, fold expansion was calculated (fold expanded). Fold expansion also varied in different patient populations, and the difference did not reach statistical significance (**Figure 5G**).

**Figure 6:**
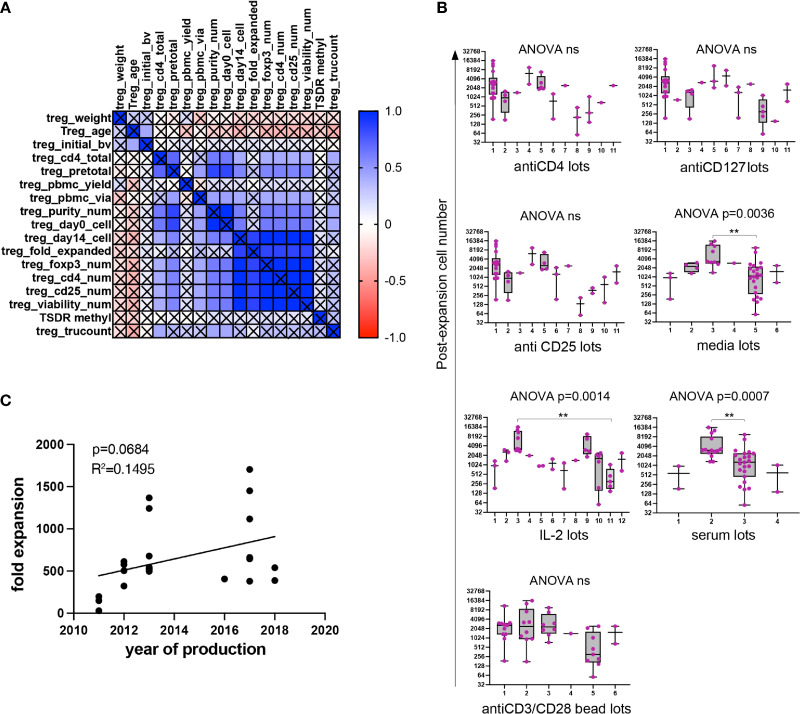
Unbiased correlation analyses. Unbiased correlation analysis of all continuous variables **(A)**. Pseudocolor; red represents negative correlation, blue represents positive correlation. Intensity of color is proportional to size of correlation. Significance threshold set at p=0.01. Black cross over cell represents nonsignificant correlation. Analysis of impact by categorical variable (material lots) on post-expansion cell number **(B)**. In box and whisker plots, the box shows mean and quartiles, whiskers show minimum and maximum values. Fold expansion by year of production among DM1 patients (n=22) **(C)**. R squared (R^2^) values and p-value assess goodness of fit, and significance of slope not equal to zero. In box and whisker plots, the box shows mean and quartiles, whiskers show minimum and maximum values. **p < 0.01. ns, not significant.

We next sought to explore potential determinants of this variability. To discover whether any independent variables (patient demographics, pre-expansion cell descriptors, manufacturing parameters) impacted manufacturing outcomes, unbiased correlation analysis was performed (**Figure 6A**). Variable definitions are found in **Supplementary Table 1**. Correlation analysis revealed several expected relationships, such as correlation between Treg numbers at the beginning and the end of the manufacturing process. Among continuous variables, the number of Treg isolated from whole blood at the beginning of the manufacturing process was significantly correlated with circulating Treg number, and with final cell yield. The impact of categorical variables on post-expansion cell number was calculated separately, which included patient demographics (sex), and manufacturing parameters (personnel, reagent lot number). Although the difference in post-expansion cell number across a few lots of reagents reached significance (**Figure 6B**), the correlations may be confounded by preferential use of lot numbers within trials. Fold expansion among type 1 diabetes patients was plotted over time and no clear correlation was seen (**Figure 6C**).

## Discussion

In this summary of manufacturing outcomes for autologous polyclonal Treg products in 7 clinical trials, we show a high rate of success in manufacturing products from different patient populations over a 10-year period. The median 434.8-fold expansion over a 14-day manufacturing period is higher than many other clinical Treg protocols that have been published (7, 13, 15, 18) This higher fold expansion may be in part due to the manufacturing protocol described here does not require rapamycin to prevent overgrowth of effector T cells. However, we did see rare manufacturing failures due to outgrowth of non-Treg cells, either CD4^+^ or CD8^+^.

Variability in total yield of Treg during the culture period significantly correlated with patient disease status and number of Treg in the patient’s peripheral blood, but showed weak or no correlation with reagent lots, manufacturing personnel, or other manufacturing parameters. This result underscores the importance of patient disease state in contributing to variability in cGMP manufacturing outcomes and the importance of including patient material, if possible, during process development of manufacturing protocols. Batch-to-batch variability observed among the reagents used in this study was small and did not have important manufacturing impacts. However, we have observed significant batch-to-batch variability during our qualification process across lots of human serum. This result underscores the importance of a formal qualification program for materials and reagents such as human serum. Although our clinical trials did not include or exclude patients based on peripheral blood Treg counts, this clinical parameter could be useful in the future for predicting manufacturing outcomes. Importantly, existing clinical assays for peripheral blood Tregs are often not comparable, so a standard assay is required to compare data between different trials or different centers. Although the age of patients did not significantly correlate with initial Treg numbers or final Treg yield, we cannot completely exclude a role for patient age in manufacturing outcomes.

Treg manufacturing cost is mostly contributed by the cost of the GMP grade materials and salary of highly skilled personnel. The costs changed over time due to increases in the costs of materials and labor. To choose a point in time relevant to this report, materials for a single Treg product in 2016 was estimated at approximately $26,500. Labor costs are much more difficult to calculate given that the manufacturing personnel performed many other duties during the period under consideration.

This study is limited by its relatively small number of Treg products. Excluding DM1, relatively few products were made in each trial. Polyclonal Treg products were not systematically evaluated for *in vitro* suppression capacity, and in-depth cell profiling was not performed. Greater insight into correlations among *in vitro* Treg expansion, *in vitro* suppression, and cellular manufacturing parameters could help guide future Treg therapy efforts.

## Data Availability Statement

The raw data supporting the conclusions of this article will be made available by the authors, without undue reservation.

## Ethics Statement

Ethical review and approval was not required for the study on human participants in accordance with the local legislation and institutional requirements. Written informed consent for participation was not required for this study in accordance with the national legislation and the institutional requirements.

## Author Contributions

JE, QT, and JAB designed the analyses. BRS designed and built the RedCap database. AP, LM, AL, LA, FK, ML, VN, WL, SP, JX, and ASL executed production and collected data. LA and BS entered data in database. JB, JE, and QT analyzed data and wrote and edited the manuscript. All authors contributed to the article and approved the submitted version.

## Funding

This study was funded by the Department of Laboratory Medicine at University of California San Francisco. The clinical trials mentioned were supported by funding from the Sean N. Parker Autoimmune Research Laboratory, JDRF, NIAID, NIDDK.

## Conflict of Interest

JAB is a co-founder, CEO and a Board member of Sonoma Biotherapeutics. He is a co-founder of Celsius Therapeutics; a member of the Board of Directors of Gilead and Provention Bio, and a member of the scientific advisory boards of Arcus Biosciences, Solid Biosciences, and Vir Biotechnology. QT is a co-founder of Sonoma Biotherapeutics.

The remaining authors declare that the research was conducted in the absence of any commercial or financial relationships that could be construed as a potential conflict of interest.

## Publisher’s Note

All claims expressed in this article are solely those of the authors and do not necessarily represent those of their affiliated organizations, or those of the publisher, the editors and the reviewers. Any product that may be evaluated in this article, or claim that may be made by its manufacturer, is not guaranteed or endorsed by the publisher.

## Supplementary Material

Supplementary Table 1 Correlation variables and units.
