# Supplementary material for: Polyclonal Regulatory T Cell Manufacturing Under cGMP: A Decade of Experience
Source: Front Immunol. 2021 Nov 18;12:744763. doi: 10.3389/fimmu.2021.744763 (PMC8636860; doi:10.3389/fimmu.2021.744763)
Supplement: Supplementary Table 1 — Correlation variables and units. [file Table_1.docx]

Supplementary Table 1: correlation variables and units

| **Parameter** | **Description** | **unit** |
| --- | --- | --- |
| treg_weight | patient weight | kilograms |
| treg_age | patient age | years |
| treg_initial_bv | initial blood volume obtained | milliliters |
| treg_CD4_total | total cell number of CD4^+^ cells | millions |
| treg_pmbc_yield | Number of peripheral blood mononuclear cells (PBMCs) recovered from peripheral blood | millions |
| treg_pbmc_via | Viable PBMC cell number ((%live * pbmc yield) | millions |
| treg_purity_num | FOXP3 expression in isolated Tregs | millions |
| treg_day0_cell | isolated Treg cell number | millions |
| treg_day14_cell | cell number post-expansion | millions |
| fold_exp | calculated expansion (post-expansion/ isolated Tregs) | (no units) |
| treg_FOXP3_num | FOXP3^+^ cell number in post-expansion cells (%FOXP3 * post-expansion cell number) | millions |
| treg_CD4_num | CD4^+^ cell number in post-expansion cells (%CD4 * post-expansion cell number) | millions |
| treg_CD25_num | CD25^+^ cell number in post-expansion cells (%CD25 * post-expansion cell number) | millions |
| treg_viability_num | post-expansion viable cell number (%live * post-expansion cell number) | millions |
| treg_TSDR | methylation status of non-coding region in *FOXP3* gene locus | % demethylated |
| treg_trucount | circulating Treg count | cells/ µL |
